# 

**Published:** 1897-08-28

**Authors:** 


### EARLY EDITION.

**Notices of Births, Deaths, and Marriages** are inserted in this column at a charge of 2s. 6d. for an announcement not exceeding four lines.

### NOTICE TO CORRESPONDENTS.

All MS., Letters, Books for Review, and other matters intended for the Editor should be addressed The Editor, "The Hospital," The Hospital Building, 28 & 29, Southampton Street, Strand, W.C.

The Editor cannot undertake to return rejected MS., even when accompanied by stamped directed envelope.

**Notice to Advertisers and Subscribers.**

All Advertisements, Orders for Copies of The Hospital, and Business Communications should be addressed to **THE MANAGER, (Not to The Editor).** The Hospital, The Hospital Building, 28 & 29, Southampton Street, Strand, London, W.C.

The Cost of Subscription, post free, is as follows:—Per week, 2½d.; per quarter, 2s. 8d.; per half-year, 5s. 3d.; per annum, 10s. 6d. Foreign:—Per annum, 15s. 2d.; payable, in all cases, in advance.

**NOTICE.—Vol. XXI. of "The Hospital" (October, 1896, to March, 1897), in handsome cloth binding, is on sale at the Office of this Journal, price 7s. 6d. Cases for binding the Numbers contained in this Volume, 1s. 6d. Index to Vol. XXI., 2½d., post free.**

**The Monthly Part for September will be ready on September 1st. Price 6d., by post 8d.**

### SCRAPS AND GLEANINGS.

During the year 1896 676 cases were treated at the Boston Insane Hospitals, or a daily average of 237 males and 260 females.

The Fishmongers' Company have sent a donation of fifty guineas to the Royal Hospital for Diseases of the Chest, City Road, E.C.

Three hundred and sixty-six in and 4,165 out patients were treated at the Yarmouth Hospital during the year ended May 31st, 1897.

Forty-six more cases were treated at the Exeter Homœopathic Dispensary in 1896 than in 1895, the numbers being 612 and 566 respectively.

The average cost of each in-patient per day at the Royal Lancaster Infirmary in 1896 is calculated by the hospital authorities at 4s. 2d., against 3s. 2d. in 1895.

The principle of the Royal Literary Fund is to administer assistance to authors who may be reduced to distress, and in carrying out this principle £2,120 was expended in grants in 1896.

There were 30 patients in the Friedenham Home for the Dying on January 1st, 1896, and 101 were admitted during the year, the average number of beds occupied being between 32 and 33.

The number of patients treated at the Lytham Cottage Hospital and Convalescent Home has sprung from 74 during the year ended March 31st, 1896, to 128 during the year ended March 31st, 1897.

Annual subscriptions at the Oldham Infirmary, which amounted to £1,388 in 1887, gradually fell off until they reached only £1,179 in 1894. In 1895, however, they had risen to £1,239, and last year (1896) to £1,319.

During the half-year ended June 30th, 1897, 706 in and 15,167 new out patients were treated at the East London Hospital for Children, compared with 800 in and 9,957 new out patients in the corresponding period of 1896.

It was stated at the annual meeting of the Dundee Royal Infirmary that in 1875 the subscribers to the hospital numbered 676, and their contributions amounted to £1,706, while in the year just ended they had fallen to 476, contributing £1,181, although the population had increased by some 40,000.

Of the 117 patients treated at the Chorley Cottage Hospital in 1896, 90 were treated free of cost, and the remaining 27 paid on an average 4s. 10d. a week. Ten out of the 13 beds were constantly occupied during the year. Eight hundred and sixty-nine persons were either visited at their own homes or treated at the dispensary.

The number of hospitals and dispensaries open in the Hyderabad assigned districts in 1896 remained the same as in the previous year, viz., 47. Four thousand five hundred in and 340,134 out patients were treated. Three hundred and twenty-two beds are provided, of which a daily average of only 129 were occupied. The expenditure amounted to 82,334 rupees.

One hundred and one dispensaries were open in the province of Assam on December 31st, 1896, compared with 97 on the previous December 31st. Five hundred and sixty-eight thousand, six hundred and ninety-six patients were treated in 1896, against 544,938 in 1895. Included in these are 6,554 and 5,987 in patients in 1896 and 1897 respectively, or an average daily number of 307, against 283 in 1895 and 284 in 1894.

### The Student of Medicine.

### APPOINTMENTS.

W. P. Simpson, M.B., C.M.Edin., appointed Medical Officer of the Pelham Lounds District of the Boston Union. J. Torrens, M.D., appointed Medical Officer to the Quatt District School, and Medical Officer and Public Vaccinator for the Claverley District of the Bridgnorth Union. W. Vernon, L.R.C.P.Edin., M.R.C.S.-Eng., appointed Medical Officer for the Bitton District of the Warmley Union. R. P. H. Whitmarsh, M.D.Brux., L.R.C.P., L.R.C.S.E., L.S.A.Lond., appointed Resident House Physician to the Hospital for Consumption and Diseases of the Chest, Brompton. James Harold Patterson, Edinburgh, appointed House Surgeon to the Huntingdon County Hospital.

### ROYAL COLLEGE OF PHYSICIANS AND SURGEONS, EDINBURGH.

The following questions were set in the examination for the triple qualification :—

Elementary Biology.—(All the questions to be answered).

1. Give a short account of the origin and mode of growth of the hypha of a mould.
2. What are the characteristics of living protoplasm ?
3. Describe the fully formed unimpregnated egg of the pigeon.
4. Give a detailed description of the frog's vertebral column, pointing out its chief difference from that of a bird.

Elementary Anatomy—Four Years' Course.—(All the questions to be answered.)

1. Give a short description of the arteries which take part in the elbow-anastomosis.
2. Describe the plantar fascia.
3. Give the insertions and nerve-supplies of the following muscles: (1) Peroneus brevis; (2) peroneus tertius; (3) extensor carpi radialis longior; (4) extensor carpi radialis brevior; (5) teres minor; (6) quadratus femoris.
4. How do the following nerves enter the forearm ? (1) Median; (2) radial; (3) ulnar.

Physics.—(All the questions to be answered.)

1. Explain the production of the colours in (*a*) mother-of-pearl, (*b*) soap bubbles, (*c*) the sky, (*d*) rainbows.
2. Upon what factors does the intensity of pressure at varying depths in a liquid depend ? Calculate the pressure of water at a depth of 1,000 feet (a cubic foot of water weighs 62·33 lbs.)
3. What is meant by the mechanical equivalent of heat, and by what means has it been determined ?
4. Describe four distinct methods of developing an electric current, and show how the presence of the current may be detected.

Chemistry.—(All the questions to be answered. Equations to be given where possible.)

1. Describe the preparation of potassium ferrocyanide. State its characteristic reactions.
2. Give the chemical names and formulæ of the following ores: "Red hematite," "cinnabar," "galena," "realgar," "calamine." Describe the process for obtaining the metal from any one of them.
3. What is meant by hardness of a water ? How and to what extent can it be removed ?
4. How is benzene usually obtained ? How may it be prepared from benzoic acid ?

